# Supplementary material for: Chromothripsis during telomere crisis is independent of NHEJ, and consistent with a replicative origin
Source: Genome Res. 2019 May;29(5):737–49. doi: 10.1101/gr.240705.118 (PMC6499312; doi:10.1101/gr.240705.118)
Supplement: Supplemental Material [file supp_gr.240705.118_Supplemental_file_1.zip › contigs/annotated_contigs/DB53/contig.4.DB53_length_1062_mean_cov_10.1920903955.docx]

**DB53_length_1062_mean_cov_10.1920903955**

```
TGGCAATTATTCAGTATTCACTAACTCAAATTTCACAAGGACTTTTTTTTTCTTCAACTCCTATTTTAAGTTCAGGGGTACATGTGCAG
 >chr8:77989337-77989756 + E=5e-237
GATGTGCAGGTTTGTCACATCAGTAAAAATGCACTATGGTGGTTTGCTGCACAGATCATCCCATCGCCTAGGTATTAAGCCCAGCATCC

ATTAGCTATTAGTCCTGATGCTCCTTCTCTTCCTGCCCCCCAACCCCAACCGGCCCCAGTGTGAGTTTTTCCCCTCCATGTGTCCATGT

GTTCTCATCATTCAGCTCCCACTTGTAAGTGAGAACATGCGATGTTTGGTTTTCTGTTCCTGCATTAGTTTGCTGAGGATAATGGCCTC

CAGCTCCATTTATGTCCCTGCAAAGGACATGAGCTCATTCCTTTTTGTGGCTGCAAAATG|TTC|TATTAGGTCTGCTTGTTGCAGAGC
                                                             >chr8:88807965-88808022 -
TGAGTTCAGATCCTGGATCTCCTTGTTAA|C|AGCCATTGGGGGAAGTATAGTAAATCTTGTCATACTCCCACTTCCAAAGCACATGTA
E=3e-22                       >chr17:68800712-68800920 - E=4e-113 p=4e-02
GAACTCATTTTTGTGTTGTTTCCTTTTTGGGGGAGTGGGAGGGGAGGAAAGATAAAAGAAAAACTCTCCTCCACCTTAAGAGTGGGGGA

TAGGACAAGAAGGAAACATTTTATAACCCAGGATCTTATACAAATACCTTCAGAATTCTGA|AGCATAA|CAGAACAGAGATAGACACA
                                                                     >chr8:80828575-808
TTCTTAGCTGTCAGTGCAGAAAGCTGAAAACCAACCTGATTTACTGCAGAATCCTCAGAATATTCAGGAACTGGCAGAACCAGGTACTT
28930 - E=1e-181
CTAGATCTCAGAGTGAAATGGGGTTAGGGGAGGTCAGAATAAAGAGATTTGGGGTCCAGCCATGGTGGCTCACGCCTGTAATCCCAGCA

CTTTGGGAGGCCAAAGTGGGCGGATTACCTGAGGTCAGGAGTTCGAGACCAGCCTTGCCAACATGGTGAAACCCCATCTCTACTAAAAA

TACAAAAACTAGCCAGGCGTGGTGGTGCACACCTGTAGTCCCAGCTACTCAGGAGGCTGAGGCAGGAG|GATTGCTTGAGCCCAGGAGG

T
```
